# Clinical Outcomes of Mechanical Prosthetic Valve Thrombosis

**DOI:** 10.7759/cureus.8760

**Published:** 2020-06-22

**Authors:** Hamid Sharif Khan, Zainab Ijaz, Muhammad Ali, Mohsin Saif, Uzma Ishaq, Ahmed Kamal, Umar Ikram, Rana Abdul Sattar, Jahanzeb Malik

**Affiliations:** 1 Cardiology, Rawalpindi Institute of Cardiology, Rawalpindi, PAK; 2 Internal Medicine, Windsor University School of Medicine, Cayon, KNA; 3 Cardiology, Armed Forces Institute of Cardiology, Rawalpindi, PAK; 4 Hematology and Medical Oncology, Fauji Foundation Hospital, Rawalpindi, PAK

**Keywords:** mechanical prosthetic valve thrombosis, fibrinolytic therapy, redo surgery, pakistan

## Abstract

Objective

To evaluate characteristics and outcomes of patients presenting with mechanical prosthetic valve thrombosis in a tertiary cardiac center in Pakistan.

Methods

This was a prospective and interventional study conducted at Rawalpindi Institute of Cardiology over a period of two years. The clinical characteristics of patients presenting with clinical suspicion of prosthetic valve thrombosis were recorded. They were, then, subjected to streptokinase, redo surgery and heparin based on their hemodynamic stability, thrombus burden and surgical risk. The patients were then followed for the outcomes of the study.

Results

Out of 576 patients with mechanical valve replacement during the study period, 70 patients had developed prosthetic valve thrombosis. Out of 70 patients, there were 41 female (58.50%) and 29 male (41.50%) participants. The mean age of the participants was 48.40±15.00 years. The overall incidence of mechanical prosthetic valve thrombosis was 12.15%. There were 30 patients (42.80%) with a suboptimal international normalized ratio (INR) and 28 patients (40.00%) were non-compliant to warfarin therapy. The overall incidence of adverse clinical outcome was 18.00%, while the overall mortality rate was 10.00%. The mortality was higher for patients who underwent redo surgery (16.60%) as compared to patients who had received fibrinolytic therapy (9.60%).

Conclusion

Poor compliance with warfarin and suboptimal INR are the important factors causing mechanical prosthetic valve. Because of lower mortality rate, fibrinolysis with streptokinase is a reasonable treatment option for mechanical prosthetic valve thrombosis.

## Introduction

In contrast to the Western countries, rheumatic valvular heart disease is the most commonly encountered valvular pathology in the developing countries. These valvular heart pathologies mostly require surgical replacement with prosthetic valves. There are a number of complications associated with mechanical prosthetic valves postoperatively. Prosthetic valve thrombosis leading to valvular obstruction is a life-threatening complication whose treatment remains controversial [1]. The incidence of mechanical prosthetic valve thrombosis varies across countries. It has an incidence of 0.6% to 6% in the left-sided valves and up to 20% in tricuspid valves [2].

Early diagnosis of mechanical prosthetic valve thrombosis followed by prompt treatment is important as delay in diagnosis and treatment can lead to increase morbidity and mortality [3]. Different factors predispose to mechanical prosthetic valve thrombosis leading to obstruction; the most common being inadequate anticoagulation therapy followed by thrombotic states like pregnancy, atrial fibrillation and ventricular dysfunction [4]. A review of the literature has shown a lack of definite guidelines for the treatment of obstructed mechanical prosthetic valve thrombosis. The various treatment options include thrombolytic therapy, intensification of anticoagulation, thrombectomy or prosthetic valve replacement [4]. These treatment options vary from patient to patient depending upon the prosthetic valve position, size of the thrombus and hemodynamic status of the patient. Intravenous anticoagulation is considered in cases of right-sided prosthetic valve thrombosis with low thrombus burden and in left-sided prosthetic valve thrombosis if the patient is hemodynamically stable [5].

In patients with left-sided prosthetic valve thrombosis with dyspnea, New York Heart Association (NYHA) class 3 and 4 either intravenous streptokinase or surgical valve replacement can be considered depending upon the surgical risk with intravenous streptokinase preferred in patients who are at a higher surgical risk. Redo surgery is an option for those patients in which the prosthetic valve has a heavy thrombus burden and the patient is hemodynamically stable enough to tolerate cardiopulmonary bypass [6].

In this study, we discuss the characteristics and outcomes of mechanical prosthetic valve thrombosis in a tertiary cardiac center in Pakistan.

## Materials and methods

This was a prospective and interventional study conducted at Rawalpindi Institute of Cardiology, Rawalpindi, Pakistan. The study had been approved by the Institutional Review Board and Ethics Committee of Rawalpindi Institute of Cardiology (IRB#RIC/DCA/125/20). The patients presenting with clinical suspicion of mechanical prosthetic valve thrombosis after mechanical valve replacement were included in the study. After informed consent had been sought, they were subjected to a battery of tests including international normalized ratio (INR) levels, transthoracic echocardiogram and fluoroscopy.

The patients were, then, divided into three groups for treatment. The patients diagnosed with right-sided and/or left-sided mechanical prosthetic valve thrombosis who were hemodynamically stable with low thrombus burden (<0.8 cm^2^) on echocardiography were treated with intravenous heparin at the dose of 80 IU/kg/hour with a target activated partial thromboplastin time (APTT) of 1.5 to 2.5 times normal [7]. The patients who were diagnosed with left-sided mechanical prosthetic valve thrombosis with heavy thrombus burden (>0.8 cm^2^) and hemodynamic instability with high surgical risk were treated with 250,000 units of intravenous streptokinase bolus followed by infusion at the rate of 100,000 units per hour for 12-24 hours. Those patients who had low surgical risk were subjected to a redo surgical valve replacement.

The clinical outcomes were defined in terms of hospital deaths, failed thrombolysis (failure of gradient to fall across the valve), complications related to thrombolysis and redo surgery. The data were analyzed using IBM Statistical Package for Social Sciences (SPSS) version 26 (IBM Corp., Armonk, NY). Frequencies and percentages were calculated for descriptive variables. Mean and standard deviation were calculated for variables such as age. Student’s t-test and chi-square analysis were carried out for comparison of continuous and categorical variables, respectively. Pairwise t-test was used to compare clinical outcomes. A p-value <0.05 was considered significant.

## Results

Out of a total of 576 patients with mechanical valve replacement during the study period, 70 patients diagnosed with prosthetic valve thrombosis were studied. There were 41 female (58.50%) and 29 male (41.50%) participants. The mean age of the participants was 48.40±15.00 years. The overall incidence of mechanical prosthetic valve thrombosis was found to be 12.15%. The clinical characteristics of these patients on admission are reported in Table 1.

**Table 1:** Clinical characteristics NYHA, New York Heart Association

Characteristics	Frequency (%)
NYHA III	45 (64.28)
NYHA IV	25 (35.71)
Systolic blood pressure less than 90 mmHg	33 (47.14)
Thrombus burden greater than 0.8 cm^2^	60 (85.71)
Thrombus burden less than 0.8 cm^2^	10 (14.28)

The distribution of the type of prosthetic valve involved in the disease process is shown in Table 2.

**Table 2:** Valves involved in thrombosis

Type of prosthetic valve thrombosis	Frequency (%)
Mitral	48 (68.57)
Aortic	20 (28.57)
Tricuspid	1 (1.43)
Mitral and aortic	1 (1.43)

There were 30 patients (42.86%) with a suboptimal INR. Non-compliance was noted in 28 patients (40.00%). In five patients (7.14%), the thrombosis was related to another underlying disease and in seven patients (10.00%), no cause could be ascertained.

Based on the clinical presentations and criteria for treatment, 52 patients (74.28%) were treated with intravenous streptokinase (fibrinolytic therapy), 12 patients (17.14%) were subjected to redo surgery and six patients (8.57%) were treated with intravenous heparin. The adverse clinical outcomes were noted for 13 patients. The outcomes are further detailed in Table 3.

**Table 3:** Adverse outcomes

Outcome	Frequency (%)
Cerebrovascular accident	3 (4.28)
Death	7 (10.00)
Failed thrombolysis	2 (2.85)
Critical limb ischemia	1 (1.42)

The patients with failed thrombolysis underwent redo surgery which proved to be successful. The mortality rate for the fibrinolysis group and redo surgery was 9.60% and 16.60%, respectively. This difference was not significant when compared using a chi-square test.

## Discussion

Mechanical prosthetic valve thrombosis is a clinical emergency which is associated with high rates of morbidity and mortality. In our study, the incidence of prosthetic valve thrombosis was 12.15%. This is in contrast to the incidence of 0.3% to 6% found in developed countries but similar to the 10% to 12% incidence rate found in developing countries [8,9]. The main reason for this high incidence is the low socioeconomic and low education status of most of the patients, which results in poor compliance of oral anticoagulation therapy. In our study, 82.86% of the patients either had suboptimal INR or poor compliance with oral warfarin therapy. Similar results were found in another study in which 55.50% of the patients had suboptimal INR and 20.90% of the patients were non-compliant with warfarin therapy [10]. The reason being female predominance of patients (58.50%) who are usually lost to follow-up after replacement resulting in suboptimal INR and poor compliance with warfarin.

The overall mortality in our study was found to be 10.00%. There are variable rates of mortality based on the degree of severity of thrombosis as well as the treatment modality involved [11]. A study had reported 82.00% as initial success rate and mortality rate of 10.00% with fibrinolysis [12]. Based on this study, it was inferred that fibrinolysis is a better option in treating left-sided mechanical valve thrombosis in whom surgical risk is high [13]. In a study of 110 patients with prosthetic valve thrombosis, streptokinase (250,000 IU intravenous over 30 minutes followed by 100,000 IU/hour infusion) was used for treatment and complete resolution of symptoms was noted in 81.80% of the patients with partial recovery in 10.00% of patients. The complications included embolic episodes (19.10%), bleeding (19.00%) and deaths (7.30%) [14]. The mortality rate with fibrinolysis in our study was 9.60% which was comparable to other studies [15,16]. The main reason for a comparatively lower mortality rate was the early diagnosis and prompt management and strict monitoring of such patients in the emergency department.

Redo surgery is the mainstay of the modality of treatment for hemodynamically stable patients and those showing increasing gradient across the valve due to pannus. The mortality rates with redo surgery differ in different studies based on the valve involved, with higher mortality rates in mitral involvement and the hemodynamic instability [17]. In our study, mortality with redo surgery was found to be 16.60%. This was a higher incidence contrast to the other studies, in which redo surgery was safer [18]. In contrast, mortality rates as high as 29.20% among patients undergoing redo surgery for prosthetic valve thrombosis have been reported as well [19]. This is explained by a failure of response to fibrinolytic therapy which increased the patients’ risk for surgery. In some patients, the mitral prosthesis was also responsible for the mortality rate. The limitations of our study include the small sample size and the single-centered nature of the study.

## Conclusions

Poor compliance with warfarin and subtherapeutic INR levels are the important factors causing mechanical prosthetic valve thrombosis, thus necessitating the need for patient education. Fibrinolysis with streptokinase is a reasonable treatment option for mechanical prosthetic valve thrombosis.
